# The Medical Law

**Published:** 1896-10

**Authors:** 


### EDITORIAL DEPARTMENT.

F. E. DANIEL, M. D., Editor.
S. E. HUDSON, M. D., Managing Editor.
A. J. SMITH. M. D., Galveston, Associate Editor.

EDITORIAL STAFF:

PROF. J. E. THOMPSON, M. D., Texas Medical College, Galveston; Surgery.
PROF. WM. KEILLER, M. D., Texas Medical College, Galveston; Obstetrics and Gynecology.
PROF. DAVID CERNA, M. D., Texas Medical College, Galveston; Therapeutics.
PROF. A. J. SMITH, M. D., Texas Medical College, Galveston; Medicine.
DR. R. H. L. BIBB, City of Mexico; Foreign Correspondent.

Published Monthly at Austin, Texas, by Drs. Daniel and Hudson. Subscription price $2.00 a year in advance.

Eastern Agents: The Monihan-Fairchild Special Agency, 24 Park Place, New York City.

Official organ of the West Texas Medical Association, the Houston District Medical Association, the Austin District Medical Society, Brazos Valley Medical Association, the Galveston County Medical Society, and several others

### THE MEDICAL LAW; A SUGGESTION FOR RELIEF.

The Medical Law in Texas appears to be a dead letter. As inefficient as it is, and as unsatisfactory, if it were *enforced* it is far better than no law at all. The statute requires that every person offering to practice medicine in Texas shall appear before a board of medical examiners—(and the law provides for a board in each congressional district)—stand examination and obtain a license; *except those having a diploma from some regularly chartered medical college*, a big enough exception, really; and those having such diploma are simply required to record it in the office of the clerk of the county court of the county where it is proposed to practice. Thus it will be seen that a person has two alternatives. He can practice on a license and no diploma, or on a diploma provided it be recorded.

The great defect in the law is—that there is no provision made for discriminating as to diplomas; no one whose business it is to see that the diplomas presented for registration emanate from "regularly chartered medical colleges," and the result is—that the holder of any kind of a diploma from any kind of college, imaginary or real—can and does practice without molestation. Another defect is, that there is no compulsion—practically—to cause non-graduates to be examined, or holders of diplomas to cause them to be registered. True—one who engages in practice without having complied with one or the other of these requirements runs the risk of prosecution; but that risk is practically *nil*. Unless some one will bring the fact that the law is not being complied with—to the knowledge of the grand jury—the offender is safe from arrest; as it is made nobody's business to see to it, and hence the law is inoperative. The reluctance on the part of the medical profession to report such violations of the law is a great hindrance and drawback, beceuse—if they will not do it no one else seems to feel called upon to do it; and hence it goes on—the law is disregarded with entire impunity. Hence—the medical practitioners—those loud in their demands for an efficient law to "regulate the practice"—are themselves to blame for the general disregard of the existing law. What reason is there to believe that a new statute—more rigid in its requirements, would be more efficient for the suppression of irregular practice than the existing one—if there is no provision made for its enforcement? The boards cannot compel persons to appear for examination and license; nor can the county clerk compel holders of diplomas to have them registered; and when offered for registration they cannot determine whether or not they emanate from "a regularly chartered medical college." The boards are authorized only to examine such applicants as voluntarily present themselves; and the clerk registers without question everything presented to him—diploma, certificate or what not; it is all fish that comes to his net—his fee is a dollar—and he takes toll of all grist that comes his way. "Anything passes muster." And—so it will be under the new law—should the association bill become a law; for nowhere in the bill are these defects provided for.

Again, as to "regularly chartered medical colleges," we know that the Mound City concern—the "Texas Health College," "the myth of the mound," was "regularly chartered," but no such college exists, or ever did exist; yet its diplomas must, perforce, under the law—pass as authority. True—when a street fakir invades a town with his band wagon, and begins to "practice medicine"—some doctor is generally ready to point out to the authorities that he is violating the law, and such persons have been arrested and fined. If a practicing physician will do this, why will he hesitate to go to the district attorney and show him that there are physicians—seemingly in good standing, practicing medicine every day—who have not complied with the formality of registering their diplomas? And others practicing who have no diploma and no license—except, perhaps, a temporary license given by a single member of some board—the date of which license has long since run out. We know of such a case,—a very prominent medical man—sufficiently so to be thought worthy of State appointment.

Under existing circumstances,—the utter disregard of the law on the part of so many who are now illegally practicing medicine in Texas—in view of the strong probability that the legislature will give us no relief; and the apparent fact that the Attorney-General will not see that county and district attorneys bring such cases of evasion of the law to the attention of the grand juries and cause the offenders to be prosecuted—the JOURNAL suggests as the only remedy available—that medical men take the matter in hand themselves and see that the law is complied with. We call upon physicians everywhere in Texas—where a case comes to their knowledge of a person who is practicing medicine in violation of law, to make it a point to report it to the grand jury, and to appear, if necessary, as a witness; it matters not what construction may be put upon his doing so. Physicians hesitate to take this step for fear of being accused of envy or jealousy of a competitor. But that is sheer nonsense. If he knew of any other law being violated he would not hesitate to report it; why should he do so in a case of violation of the medical practice act? In the law it is a crime to conceal a crime. Is not this action on the part of the only persons interested practically condoning if not concealing—a crime?

The JOURNAL believes that if the regular practitioners of medicine all over Texas will agree to co-operate, and pledge themselves to take active steps in enforcing the law—and will carry on a vigorous campaign against offenders—the existing statute can be made much more efficient, and will answer very well until the legislature can be made to appreciate the magnitude of the danger of the quack doctor to the public health, and to give the profession the long-sought and urgently needed relief. It will involve neither trouble nor expense to carry out this suggestion. When a doctor is known to be practicing in violation of the law, let some leading physician go to the grand jury and give the information. Witnesses will not be necessary. Reference to the books of the clerk of the county court will show—prima facia—that the accused has not complied with the law. The grand jury will then be compelled—if they do their duty—to return an indictment. In nearly every town there are such cases. Who, nów, will take the initiative? Three cases in Bell county have been reported to the JOURNAL; several in Jefferson, several in Fannin; in fact, reports of violations of this act, by men in full practice, come to us every day—as if the "Red Back" were in position to give the desired relief. We have, in every instance, reported the offenders to the Attorney-General. We have asked him to issue a circular letter to the judges of the several districts instructing them to order the grand jury to investigate the matter in every section, and he has promised us that he would do so. That is all we can do; meantime we urge upon our readers the necessity of taking up the cudgel in their own defense and protection, and where no one else will report offenders to do so themselves, without hesitation, fear or favor.

